# Neurovascular Unit: A critical role in ischemic stroke

**DOI:** 10.1111/cns.13561

**Published:** 2021-01-02

**Authors:** Liyun Wang, Xiaoxing Xiong, Luyuan Zhang, Jian Shen

**Affiliations:** ^1^ Department of Neurosurgery Shengzhou People’s Hospital (the First Affiliated Hospital of Zhejiang University Shengzhou Branch) Shengzhou China; ^2^ Department of Neurosurgery Renmin Hospital of Wuhan University Wuhan China; ^3^ Department of Neurosurgery First Affiliated Hospital School of Medicine Zhejiang University Hangzhou China

**Keywords:** Ischemia, MicroRNAs, Neurovascular unit, Stroke, Therapy

## Abstract

Ischemic stroke (IS), a common cerebrovascular disease, results from a sudden blockage of a blood vessel in the brain, thereby restricting blood supply to the area in question, and making a significantly negative impact on human health. Unfortunately, current treatments, that are mainly based on a recanalization of occluded blood vessels, are insufficient or inaccessible to many stroke patients. Recently, the profound influence of the neurovascular unit (NVU) on recanalization and the prognosis of IS have become better understood; in‐depth studies of the NVU have also provided novel approaches for IS treatment. In this article, we review the intimate connections between the changes in the NVU and IS outcomes, and discuss possible new management strategies having practical significance to IS. We discuss the concept of the NVU, as well as its roles in IS blood‐brain barrier regulation, cell preservation, inflammatory immune response, and neurovascular repair. Besides, we also summarize the influence of noncoding RNAs in NVU, and IS therapies targeting the NVU. We conclude that both the pathophysiological and neurovascular repair processes of IS are strongly associated with the homeostatic state of the NVU and that further research into therapies directed at the NVU could expand the range of treatments available for IS.

## Graphical abstract

Neurovascular unit plays a leading role in the pathophysiological process of ischemic stroke. Both cell‐based therapies and drugs targeting the neurovascular unit can fight against ischemic stroke. The entire multiple cells’ interaction framework of the neurovascular unit are worth to further study to explore the therapeutic potential of the neurovascular unit in clinical.

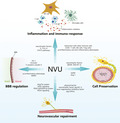

## 1 BACKGROUND

Stroke is the leading cause of disability, as well as the second leading cause of death worldwide.^1^, ^2^, ^3^, ^4^, ^5^ Ischemic stroke (IS), in particular, accounts for 85% of strokes.^6^ Due to the death of brain cells following the permanent or transient blockage of blood vessels, IS imposes a heavy economic and health burden on society.^4^, ^7^ Following the failure of several decades of large scale neuroprotective clinical trials, the focus of stroke treatment shifted from a neuroprotective approach to neurovascular protection.^8^, ^9^ The concept of the neurovascular unit (NVU), comprised of neurons, astrocytes, smooth muscle cells (SMCs), endothelial cells (ECs), pericytes (PCs), and the basal lamina matrix, emphasizes the unique cross talk between neurons and the cerebral vasculature, and ultimately, the pivotal role the NVU plays in IS progression.^10^ With effective reperfusion strategies implemented, intravenous thrombolysis and thrombectomy have become the most common treatments administered to IS patients.^11^ Nowadays, the usage of tissue‐type plasminogen activator (tPA) consists a widely accepted treatment, that is most effective when administered within 4.5 h after acute ischemic stroke (AIS).^12^, ^13^, ^14^ Unfortunately, it is only applicable to a limited number of patients because of the strict time window of tPA treatment. Thus, the provision of other effective treatments is urgently required.^13^

In this review, we discuss the effects of the NVU on blood‐brain barrier (BBB) regulation, cell preservation, inflammatory immune response, and neurovascular repair during or after IS, as well as the regulation of the NVU by noncoding RNAs (ncRNAs). Lastly, we review existing therapeutic approaches and prospects for IS treatments targeting the NVU.

## 2 THE CONCEPT OF THE NEUROVASCULAR UNIT

The neurovascular unit (NVU), a groundbreaking concept consisting of multiple components, includes neurons, glial cells, vascular cells (endothelial cells or ECs, pericytes or PCs, and smooth muscle cells or SMCs), and the basal lamina matrix within the brain vasculature.^15^, ^16^ The concept emerged from the first Stroke Progress Review Group meeting of the National Institute of Neurological Disorders and Stroke of the National Institutes of Health represents a conceptual framework incorporating neurons and the adjacent vasculature.^10^

It is now recognized that the interactions between various components of the NVU are highly important.^15^ The BBB and cerebral blood flow (CBF) are precisely controlled by the NVU, thus maintaining a homeostatic brain microenvironment.^16^, ^17^ Endothelial cells form a highly specialized membrane around blood vessels.^17^ Pericytes in the central nervous system (CNS) contribute to both neurogenesis and vasculogenesis, and PCs localized within blood vessels may act as multipotent vascular stem cells.^17^, ^18^, ^19^, ^20^, ^21^, ^22^ The loss of PCs leads to reduced expression of specific tight junction (TJ) proteins and subsequent BBB disruption.^23^ Astrocytes extend end feet to PCs and SMCs to regulate their constriction and relaxation, thereby adjusting CBF.^24^, ^25^ Astrocytes also regulate the balance of synaptic glutamate partly via Ca^2+^ oscillations as a timely response to changes in ions and metabolism in neuronal cells.^25^ Although the classic definition of NVU does not include microglia and oligodendrocytes, structurally and functionally they are closely related to the NVU. Oligodendrocytes not only produce neurotrophic factors, but also form myelin sheaths that support the transmission of action potentials.^26^ Furthermore, they may also serve as antigen‐presenting cells.^27^ As immune cells in the CNS, microglia can modulate the innate immunity of astrocytes by releasing various signaling molecules.^28^, ^29^ In summary, all the NVU components are closely related in structure, and integral in function to preserve brain homeostasis.

## 3 THE ROLES OF THE NVU IN IS

The pathophysiological process of IS consists of three stages in time and space: a) the hyperacute phase (minutes to 6 h); b) the acute and subacute phase (hours to 7 d); and c) the chronic phase. During the course of injury and inflammation, endogenous protective and repair mechanisms are activated simultaneously, and the ratio of these activities determines the outcome of IS.^30^ The roles NVU plays in IS are crucial, which we summarize in four parts: BBB regulation, cell preservation, inflammatory immune response, and repair during or after IS.

### 3.1 BBB regulation during IS

The function of the BBB depends on the TJs between ECs and the perivascular microenvironment. In the acute phase following the initiation of ischemia, NVU dysfunction directly promotes the breakdown of the BBB. For example, the reduction of expression of certain proteins (such as occludin, claudin‐5, and ZO‐1) enhances BBB permeability and increases the risk of inducing vasogenic cerebral edema.^30^, ^31^ In addition, PCs further promote the development of cerebral edema by transforming into a proinflammatory phenotype.^30^ Glial cells may contribute to BBB destruction via matrix metalloproteinases (MMPs), such as MMP‐9, which digest BBB matrix proteins.^32^, ^33^ To date, tPA is the only therapeutic agent that has been approved for the treatment of patients with AIS.^12^, ^34^, ^35^ However, tPA itself activates MMPs, further exacerbating the destruction of the BBB, which not only promotes the development of neuroinflammation and edema, but also increases the risk of cerebral hemorrhage in patients treated with thrombolysis.^36^, ^37^

Therefore, in order to prevent the further development of IS, BBB protection must be a top priority; BBB repair can assist with the treatment of IS. Perlecan is a major protein of the basement membrane, with upregulated expression after IS in mice. The core protein of Perlecan called DV attaches to PC and EC, and promotes pericyte migration through the integral protein α5β1 via PDGFRβ signaling, subsequently regulating BBB repair.^38^ The permeability of the BBB is increased in mice through CLEC14A knockdown ECs, in which TJ proteins are attenuated.^39^ The PDGFR‐β signaling also regulates the recruitment of PCs into injury lesions to promote BBB recovery.^40^

### 3.2 Cell preservation

Endothelial cells are the first to be damaged in ischemic brain regions. The integrity of the TJs between ECs can be enhanced by PCs via the secretion of glial cell‐derived neurotrophic factor (GDNF) and angiopoietin‐1 (Ang‐1), which ultimately protects ECs from necrosis.^41^ Pretreatment with neutralizing antibodies of Ang‐1 blocks the PC‐induced upregulation of TJ proteins.^41^

A variety of neurotrophic factors are expressed by pericytes, including the brain‐derived neurotrophic factor, nerve growth factor, and neurotrophin‐3, which provide neuroprotective effects and facilitate neuronal and axonal regeneration in response to IS.^42^, ^43^ After pericyte ablation with diphtheria toxin, the loss of pleiotrophin, a pericyte‐secreted growth factor enriched in PCs to provide neurotrophic support, leads to both rapid neuron and CBF loss, and results in BBB damage in mice.^44^ Astrocyte‐specific Swell1 deletion mice exhibited remissive glutamate‐dependent neuronal excitability and brain damage after IS.^45^ Additionally, reactive astrocytes restrict neuronal migration toward the IS brain lesion through direct contact, while new neurons positioned close to the lesion promote functional recovery via increased Slit‐Robo signaling.^46^

### 3.3 Inflammatory immune response

Glial cells are key components of the CNS.^47^, ^48^ At the onset of stroke, astrocytes are activated immediately by molecules released from the site of injury.^49^ They secrete proinflammatory factors and MMPs that destroy the BBB, as well as neurotrophic factors that protect ischemic sites.^49^ In cerebral ischemia‐reperfusion mouse models, the overexpression of IL‐15 in astrocytes enhances the effector functions of CD8 + T and NK cells, and thus aggravates ischemic brain injury.^50^ In response to stroke, the number of Treg cells and astrocyte‐derived levels of IL‐33 and CCL1 increase. Elevated levels of amphiregulin secreted by Treg cells further regulate the IL‐6 and STAT3 pathways, thereby improving neurological functional defects.^51^, ^52^

Microglia also display both pro‐ and anti‐inflammatory phenotypes (named M1 and M2, respectively) and respond rapidly to ischemia during IS.^53^, ^54^ Within one day after IS, the proliferation and activation of microglia induced a strong inflammatory response (upregulation of TNF, IL‐1β, and IL‐6), causing severe damage to the CNS.^51^, ^55^ Protective cytokines, such as neurotrophic IGF‐1, are secreted by microglia cells several days after the onset of IS, contributing to nerve repair and survival.^56^ The inhibition of microglia activation by complement inhibitors can protect stressed neurons and reduce neuroinflammation in a mice model.^57^ On the contrary, if treatment with CSF1R antagonist reduces microglia and increases the number of neutrophils, the brain damage in mice IS brain tissue becomes even more serious.^58^ Interferon regulatory factors (IRF) are regulators of macrophage activation. The downregulation of IRF4 leads to the increased expression of IRF5, which in turn enhances the activation of M1, leading to enhanced proinflammatory response and poor stroke prognosis. On the contrary, the decrease of IRF5 helps to enhance M2 activation, inhibits the proinflammatory response, and aids functional recovery.^59^ Activated microglia and their fragmented mitochondria induce astrocyte transformation into reactive A1 astrocytes.^60^, ^61^ These lack most of the normal astrocyte functions and are neurotoxic to neurons and maturely differentiated oligodendrocytes.^60^ Interestingly, astrocytes also secrete the cytokine interleukin‐33, which in turn promotes microglial synaptic remodeling.^62^

### 3.4 Neurovascular repair

Following the acute phase of IS, the inflammatory response in the infarcted area starts to decrease and tissue repair begins to intensify. Although reactive glial cells are harmful in the early stage, reactive astrocytes also play a role as a phagocytic phenotype in engulfing cell debris to aid the recovery of brain damage via the ABCA1‐mediated pathway.^46^ The subsequently formed glial scars may still hinder the axonal bud bulging through the extensive expression of axon regeneration inhibitors (such as chondroitin sulfate proteoglycans). On the other hand, normal brain tissue is isolated from the damaged area to minimize the magnification of lesions and inflammation.^63^ The morphology of astrocytes after 2 h of transient middle cerebral artery occlusion in mice showed that the deterioration of astrocyte ultrastructure is much slower than that of neurons, indicating that, under ischemic conditions, astrocytes are more resistant to injury than adjacent neurons.^64^ In the absence of astrocytes, glutamate neurotoxicity occurs at lower concentrations in the cerebral cortex of mice.^65^

**FIGURE 1:**
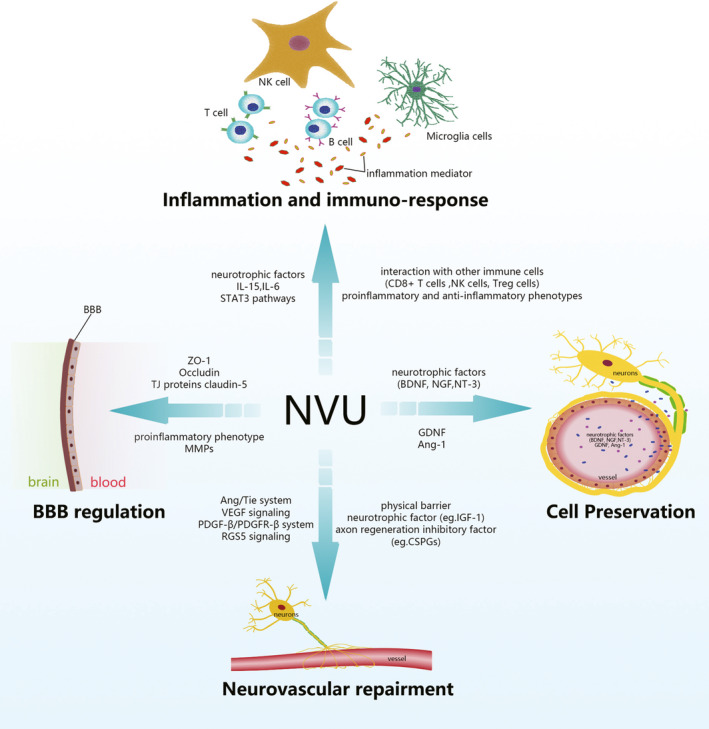
The roles of the neurovascular unit in ischemic stroke

Pericytes serve as critical regulators during angiogenesis after IS via various signaling pathways, including the Ang/Tie system, VEGF signaling, the PDGF‐β/PDGFR‐β system, and RGS5 signaling.^66^ The upregulation of ephrinB2 acted beneficially on neurovascular repair after IS by increasing pericyte recruitment and endothelial‐pericyte cell interaction. On the contrary, the inhibition of ephrinB2 expression in ECs or PCs leads to worse outcomes.^67^ Furthermore, the vascular endothelial growth factor isoform‐B also stimulates neurovascular repair following IS by promoting the function of PCs via VEGFR‐1.^68^ After a reasonable decline of pericyte‐derived fibrosis, the number of raphespinal and corticospinal tract axons was increased, which was proportional to the degree of functional recovery after CNS injury.^69^ (Figure 1).

In summary, the NVU is able to regulate BBB integrity, cell preservation, inflammatory immune response, and repair during or after IS. It secrets a variety of proteins to prevent BBB from breakdown and to promote its functional recovery. The integrity of the NVU provides strong support to cell preservation through a subtle regulation of neurotrophic factors and subsequent signaling pathways. Interestingly, it plays its critical role as a two‐edged sword in inflammatory immune responses to IS, with both the pro‐ and anti‐inflammatory phenotypes of the NVU responding rapidly to ischemia. The inhibition of microglia activation can protect the CNS from microglia related inflammatory immune response; however, the use of antagonists inhibiting microglia activation will block subsequent functional recovery. The increased recruitment of PCs and ECs is beneficial to neurovascular repair after IS, while a reasonable decrease of pericyte‐derived fibrosis promotes the outcome of CNS injury.

## 4 REGULATION OF THE NVU BY NCRNAS IN IS

Mechanisms through which NVU plays a role in IS are highly complicated, with several genes involved in relevant regulatory pathways. In addition to the large number of immuno‐inflammatory molecules discussed above, a growing number of studies have begun to recognize the important roles ncRNAs play in NVU, though no relevant work to summarize these roles exists in this regard. Therefore, a discussion on the involvement of ncRNAs in IS through NVU is detailed herein, which helps to deepen the understanding of the pathophysiological process of IS, thereby opening up possible new directions for treatment.^30^

Firstly, microRNAs (miRNAs) regulate gene or protein expression by inhibiting translation.^70^ In an established ischemia/reperfusion (I/R) rat model, exosomal mir‐26b‐5p inhibits M1 polarization of microglia via targeting CH25H and inactivating the TLR pathway, leading to reduced nerve injury after cerebral I/R. A reduction of exosomal mir‐26b‐5p has the opposite effect.^71^ Recently, it was shown that expression levels of inflammatory cytokines elevate, whereas anti‐inflammatory cytokines (IL‐4, IL‐10) and mir‐30d‐5p decrease in AIS patients compared with normal control. In addition, adipose‐derived stem cell‐derived exosomes enriched with mir‐30d‐5p have a protective effect on AIS via autophagy‐mediated microglia M1 polarization reduction.^72^

Long noncoding RNAs (lncRNAs) serve as miRNA sponges or antagomirs.^73^ They contribute greatly to the pathophysiological process of IS, for example, the lncRNA Malat1 is capable of sponging mir‐26b, mir‐30a, mir‐145, mir‐205‐3p, and mir‐200c‐3p from protecting cerebral microvascular endothelial cells, and attenuating neuronal cell death following IS.^74^, ^75^, ^76^ The expression of lncRNA Nespas is significantly upregulated after IS in an MCAO mice model. While the silencing of Nespas accelerates the apoptosis of microglia, increased Nespas expression alleviates ischemic brain lesion by inhibiting NF‐κB activation and TRIM8‐induced K63‐linked polyubiquitination of TAK1 in microglia.^77^

Emerging evidence suggests that multiple circular RNAs (circRNAs) serve as novel biomarkers and important regulators, or even triggers in various cancers.^78^, ^79^, ^80^, ^81^ Using circRNA microarray and genome‐wide bioinformatic analysis to study ischemic responses in mice subjected to transient middle cerebral artery occlusion (tMCAO) and to plasma samples from AIS patients, Han *et al* reported that circhectd1, a mir‐142 sponge, downregulates mir‐142 activity and thus leads to lower TCDD inducible poly[ADP‐ribose] polymerase (TIPARP) expression. This subsequently results in the inhibition of astrocyte activation via autophagy, while the downregulation of circHectd1 expression decreases infarct areas. In addition, circhectd1 is expressed at higher levels in AIS tissues and plasma than in control samples. These findings indicate that circhectd1 could be used as a novel type of biomarker and potential therapeutic target for IS.^82^ Additional, relevant studies are summarized in Table 1.

**TABLE 1:** The regulation of the NVU by ncRNAs in IS

NVU components	ncRNAs	Regulated Molecules /Pathways	Effect	References
Astrocytes	Mir‑29b	AQP4	Protection against ischemia‐reperfusion injury	^112^
	Mir‐133b	TGF‐β (mir‐206/RABEPK)	Regulation of neurovascular plasticity	^113^
Endothelial cells (ECs)	Mir‐27b	AMPK	Regulation of tube formation and migration	^114^
	Mir‐383	Peroxisome proliferator‐activated receptor gamma	Promotion of neurotrophy and inhibition of abnormal apoptosis	^115^
	Mir‐140‐5p	Vascular endothelial growth factor A (VEGFA)	Cell proliferation, migration and tube formation	^116^
	Mir‐155	TGF‐β/BMP, SMAD5, mTOR, NO	Improvement of CBF and supporting microvasculature	^117^
	Mir‐107	Dicer‐1	Angiogenesis	^118^
	Mir‐24‐1‐5p	HIF‐1α	Angiogenesis	^119^
	Mir‐191	NF‐Κb	Angiogenesis	^120^
	Mir‐181a	IL‐6/TNF‐α	Inhibition of the oxidized low‐density lipoprotein (ox‐LDL)‐induced immune inflammatory response	^121^
	Mir‐126‐3p/‐5p	IL‐1β, TNF‐α, VCAM‐1, E‐selectin	Maintenance of BBB integrity	^122^
	Mir‐194‐ 1	TGF‐β/SMAD	Reduction of the inflammatory response and EC permeability	^123^
Neurons	Mir‐106b‐5p	Mcl‐1/Bcl‐2	Inhibition of apoptosis and oxidative stress	^124^
	Mir‐149‐5p	P53/Caspase‐3	Regulation of cell survival and apoptosis	^125^
	Mir‐455	TRAF3	Inhibition of neuronal cell death	^126^
	Mir‐365	OXR1	Activation of antioxidant signals	^127^
Neurons, Astrocytes	Mir‐19a‐3p	Polyclonal Antibody to Adiponectin Receptor 2 (ADIPOR2)	Modulation of glucose metabolism and neuronal apoptosis	^128^
Microglia	Mir‐124	Increase of M2‐like polarized microglia number	Neuroprotection and functional improvement	^129^
	Mir‐26b‐5p	TLR	Regulation of microglia M1 polarization	^71^
	Mir‐30d‐5p	Autophagy	Regulation of microglia M1 polarization	^72^
Endothelial cells (ECs)	LncRNA‐H19	NF‐κB	Inhibition of EC apoptosis in the ASO model	^130^
	Malat1	Mir‐26b, mir‐30a, mir‐145, mir‐205‐3p and mir‐200c‐3p sponge	Protection of the NVU	74, 75, 76
Vascular smooth muscle cells (SMCs)	LncRNA‐MEG3	ABCA1	Regulation of proliferation and apoptosis in VSMCs	^131^
	LncRNA‐BANCR	NK	Facilitation of SMC proliferation and migration	^132^
Astrocytes	CircHECTD1	Mir‐142 sponge	Inhibition of astrocytic activation via autophagy	^82^
Pericytes (PCs)	CPWWP2A	Mir‐579 sponge, angiopoietin 1, occludin and SIRT1	Decrease in vascular dysfunction	^133^

## 5 IS THERAPIES TARGETING THE NVU

The aims of the current review are to overcome the limitations of the existing treatment strategies for IS and pursue faster recovery times and better recovery results. Three treatment methods targeting the NVU are summarized here cell‐based therapies, neuronal regeneration, and NVU protection.

### 5.1 Cell‐based therapies

Cell‐based therapy is an exciting emergent approach. Results of studies demonstrating that bone marrow stromal cells (MSCs) work well for promoting positive outcomes in IS models are promising.^83^ Indeed, the exogenous transplantation of MSCs initiates the repair steps of angiogenesis, axonal remodeling, and synaptic formation. The expression of neurotrophic factors is stimulated by MSCs in astrocytes, thereby enhancing neuron survival and the expression of Cx43, which promotes the gap junction of astrocytes.^84^ Furthermore, the inhibition of nerve scar formation by MSC transplantation after stroke may also promote axonal regeneration, thus enhancing the capacity for nerve repair.^85^ MicroRNAs in the miR‐17‐92 cluster, which are enriched in exosomes derived from MSCs, accelerate the reconstruction of axon‐myelin and thus recovery from IS.^86^ Many types of stem cells, including but not limited to adipose stem cells (ADSC), MSCs and pluripotent stem cells, can differentiate into functional PCs.^87^, ^88^, ^89^ However, the issue of how to transfer sufficient numbers of functional transplanted cells to specific sites remains to be addressed. In this aspect, a scaffold‐free cell sheet has been used to transplant sufficient numbers of allogeneic adipose‐derived mesenchymal stem cells in a rat stroke model.^90^ Transplanted stem cells not only replace dead neurons, but also secrete a variety of nutritional and growth factors to promote NVU regeneration and repair.^91^ Unfortunately, even though the transplantation of the stem cells has been achieved successfully, their subsequent survival, proliferation, migration, and differentiation still encounter a series of challenges.^91^

### 5.2 Neuronal regeneration

Neuroplasticity influences rehabilitation and recovery of the injury site affected by stroke. Cultured human cortical astrocytes transplanted into mice have been reprogrammed into functional neurons through retroviral expression of NeuroD1.^92^ Endothelial progenitor cells (EPCs) secrete growth factors, including FGF‐b, VEGF, and PDGF‐BB, into cell‐free conditioned media (CM). This was utilized in an IS mouse model, where both angiogenesis and neurogenesis are enhanced in mice treated with CM rich in growth factors from EPC cultures.^93^ In the infarct region of brain, Caveolin‐1 (Cav‐1) upregulates to accelerate neovascularization in wild‐type mice, while Cav‐1 knockout mice display the inverse effects.^94^ Electrical stimulation (ES) based on nanomaterials has a positive effect on the fate of neural stem cells (NSCs) in vitro. Hence, ES treatment could be a potential complementary noninvasive therapy during NSCs transplantation.^95^, ^96^ Moreover, the combination of ADSC, sodium ferulate, and n‐butylidenephthalide yields improve neovascularization and neurogenesis compared with single stem cell treatment.^97^

### 5.3 NVU protection

Reperfusion injury further leads to deterioration following IS.^98^ With pretreatment of 4‐methoxybenzyl alcohol in rats subjected to reperfusion injury, the ratio of surviving neurons increases compared with controlled groups via the regulation of Bcl‐2, caspase‐3, and Bax, while the ultrastructure of glial cells is significantly protected.^99^ β‐Caryophyllene maintains BBB integrity and prevents neuronal apoptosis by reducing proinflammatory factors and oxidative stress damage.^100^ Pericytes, though, are more vulnerable than neurons in an ischemic environment.^101^ Mitochondrial metabolism in astrocytes is enhanced by the purinergic ligand 2‐methylthioladenosine 5’ diphosphate *via* increased inositol trisphosphate‐dependent Ca^2+^ release, which provides protective benefits from IS.^102^ After tert‐butylhydroquinone treatment following permanent distal middle cerebral artery occlusion in mice, the activation of astrocytes, and angiogenesis are significantly enhanced.^103^ Cilostazol is a commonly used antiplatelet drug, which prevents the pathological detachment of astrocyte foot processes or PCs and also stimulates VEGFR2 expression and PC proliferation, thereby protecting the NVU integrity and promoting neurovascular protection.^104^ Notch‐Jagged signaling in astrocytes is increased in selegiline‐treated MCAO rats compared with control, helping to preserve the capillary network after IS.^105^ Treatment with tPA inhibits the secretion of glial cell‐derived trophic factors and damages PCs, but edaravone can reverse the damage, and maintains NVU integrity after tPA treatment.^106^ In a mouse model where ECs, SMCs, and PCs partly lack guanylyl cyclase B, the endothelial C‐type natriuretic peptide acts on PCs, thereby regulating microcirculatory flow and blood pressure.^107^ Additionally, teriflunomide improves pericyte coverage and survival, resulting in decreased TJ protein breakdown and BBB leakage.^108^ A recent study has shown that novel interpericyte tunneling nanotubes could build a functional network to regulate neurovascular coupling through linking two separate PCs on different capillaries in the mouse retina.^109^

Under hypothermic conditions, the isolation between basement membrane and pericytes is not observable at the ultrastructural level, indicating a well‐preserved BBB.^110^ Hypothermia can be attained in mice by HPI‐201 injection. Following severe stroke, HPI‐201 treated C57BL/6 mice recover with lower neurological severity scores, decreased expression levels of inflammatory factors, higher BBB integrity, and more complete conservation of the NVU compared with the controls.^111^ Thus, hypothermic protection could be a potential method to protect the NVU from IS.

## 6 FINAL REMARKS AND CONCLUSION

Undoubtedly, the NVU plays a leading role in the pathophysiological process of IS, with profound effects on the BBB, cell preservation, inflammatory immune response, and neurovascular repair. Both cell‐based and pharmacological therapies targeting the NVU can fight against deleterious outcomes following an ischemic stroke. The IS therapeutic philosophy has moved from the neuronal era to the neurovascular era; therefore, we must consider the entire framework of the NVU and conduct thorough investigations on the multiple interactions between its cells to further explore the therapeutic potential of the NVU in clinical settings.

## CONFLICTS OF INTEREST

The authors declare no conflict of interest.

## AUTHORS’ CONTRIBUTIONS

LY Wang and LY Zhang read literatures and prepare the manuscript; Chao Zhang collect literatures; XX Xiong and Jian Shen prepare the manuscript.

## Data Availability

The data that support the findings of this study are available from the corresponding author upon reasonable request.
